# Minireview: Chromatin-based regulation of iron homeostasis in plants

**DOI:** 10.3389/fpls.2022.959840

**Published:** 2022-09-16

**Authors:** Justin Su, Zhujun Yao, Yixuan Wu, Joohyun Lee, Jeeyon Jeong

**Affiliations:** ^1^Department of Biology, Amherst College, Amherst, MA, United States; ^2^Division of Natural and Applied Sciences, Duke Kunshan University, Kunshan, China

**Keywords:** iron, chromatin, histone modification, DNA methylation, nutrition, epigenetics

## Abstract

Plants utilize delicate mechanisms to effectively respond to changes in the availability of nutrients such as iron. The responses to iron status involve controlling gene expression at multiple levels. The regulation of iron deficiency response by a network of transcriptional regulators has been extensively studied and recent research has shed light on post-translational control of iron homeostasis. Although not as considerably investigated, an increasing number of studies suggest that histone modification and DNA methylation play critical roles during iron deficiency and contribute to fine-tuning iron homeostasis in plants. This review will focus on the current understanding of chromatin-based regulation on iron homeostasis in plants highlighting recent studies in Arabidopsis and rice. Understanding iron homeostasis in plants is vital, as it is not only relevant to fundamental biological questions, but also to agriculture, biofortification, and human health. A comprehensive overview of the effect and mechanism of chromatin-based regulation in response to iron status will ultimately provide critical insights in elucidating the complexities of iron homeostasis and contribute to improving iron nutrition in plants.

## Introduction

Plants evolved complex regulatory mechanisms to cope with changes in the environment, including nutrient availability (Secco et al., 2017). At the molecular level, plants respond to nutritional status by modulating gene expression at multiple levels through a network of transcription factors and *via* post-translational regulation. Multiple studies have also revealed that changes in chromatin state by histone modification or DNA methylation play important roles in nutrient homeostasis in plants (Secco et al., 2017; Séré and Martin, 2020).

Post-translational modification of histone and DNA methylation lead to transcriptional regulation by altering chromatin packaging and chemical properties of the nucleosome surface, both of which influence association of DNA-binding transcriptional regulators (Berger, 2007). Emerging evidence reveals the importance of chromatin regulation in nutritional homeostasis in plants. For example, phosphate starvation-induced genes are regulated by the histone acetyltransferase GCN5 (Wang T. et al., 2019) and histone deacetylases HDA19 and HDC1 (Chen et al., 2015; Xu et al., 2020). Histone 3 lysine 4 trimethylation (H3K4me3) was also shown to regulate gene expression under phosphate deficiency (Chandrika et al., 2013a,b). In high nitrogen, increased H3K27me3 deposition, and decreased H3K4me3 and H3K36me3 contribute to the repression of the high affinity nitrate transporter gene, *AtNRT2.1* (Widiez et al., 2011). H3K27me3 also modulates *AtNRT2.1* by limiting its induction under low nitrogen (Bellegarde et al., 2018). Multiple genes involved in sulfate uptake and assimilation are direct targets of histone methylation and acetylation (Huang et al., 2019). In addition, global changes in DNA methylation were observed under phosphate starvation (Yong-Villalobos et al., 2015; Secco et al., 2017), sulfur deficiency (Huang et al., 2016), and zinc deficiency (Chen et al., 2018). Chromatin remodeling genes were differentially expressed upon zinc or iron treatment, implying chromatin-level responses to maintain mineral homeostasis (Darbani et al., 2015). Although chromatin remodeling has not been extensively studied in the context of metal homeostasis, reports increasingly suggest the involvement of histone modification and DNA methylation in regulating iron. This minireview will focus on the current knowledge of chromatin-based regulation of iron homeostasis in plants.

Iron is an essential micronutrient for plant growth and development. Iron is an indispensable cofactor in vital metabolic processes, but improperly regulated iron causes cytotoxicity by facilitating the generation of reactive oxygen species (ROS) (Halliwell and Gutteridge, 1992). Despite being abundant in the soil, iron is not readily accessible for plants, as it is highly insoluble in aerobic conditions at neutral or alkaline pH (Colombo et al., 2014). Iron’s importance as an essential micronutrient with low bioavailability and its potential for toxicity necessitates a tightly regulated system of iron acquisition and regulation in plants. Understanding iron homeostasis is important to answer fundamental biological questions, but also to improve agriculture and human health.

## Iron deficiency response and iron uptake

In response to iron deficiency, plants induce iron uptake mechanisms that involve reducing or chelating iron (Connorton et al., 2017; Riaz and Guerinot, 2021). Dicots acquire iron *via* a reduction-based process known as Strategy I, which involves proton efflux to the rhizosphere by proton ATPases such as AHA2 to solubilize ferric chelates (Santi et al., 2005), coumarin secretion to facilitate iron mobilization (Clemens and Weber, 2016), reduction of ferric chelates to ferrous iron by FERRIC REDUCTASE OXIDASE 2 (FRO2) (Robinson et al., 1999), and ferrous iron import into root epidermal cells by IRON-REGULATED TRANSPORTER 1 (IRT1) (Eide et al., 1996). IRT1, FRO2, and AHA2 co-localize in interactomes, which likely optimize iron uptake (Martín-Barranco et al., 2020). Grasses use a chelation-based process or Strategy II for iron uptake. When iron is limited, phytosiderophores, mugineic acid (MA) and its derivatives, are synthesized (Mori and Nishizawa, 1987; Shojima et al., 1990) and secreted into the rhizosphere by Transporter of Mugineic acid family phytosiderophores 1 (TOM1) to chelate iron (Nozoye et al., 2011). Fe^3+^-phytosiderophore complexes are then transported into the root epidermal cells by the Yellow Stripe (YS) family transporters (Curie et al., 2001). Even though grasses are considered as Strategy II plants, Strategy I is used or its components exist in graminaceous plants (Bughio et al., 2002; Ishimaru et al., 2006; Cheng et al., 2007; Bashir et al., 2011; Li et al., 2016; Kaur et al., 2019; Wairich et al., 2019; Wang M. et al., 2019).

## Regulation of iron deficiency response

Responses to iron availability are controlled from transcriptional to post-translational levels (Vélez-Bermúdez and Schmidt, 2022). In particular, the complex network of basic helix-loop-helix (bHLH) family transcription factors involved in iron deficiency response has been extensively studied (Gao et al., 2020; Schwarz and Bauer, 2020). In Arabidopsis, FER-LIKE IRON DEFICIENCY-INDUCED TRANSCRIPTION FACTOR (FIT)/bHLH29 directly regulates *IRT1*, *FRO2*, *FIT*, and other genes involved in iron uptake under iron deficiency (Colangelo and Guerinot, 2004; Jakoby et al., 2004; Yuan et al., 2005). FIT forms heterodimers with subgroup Ib bHLH transcription factors, bHLH038/39/100/101, to activate FIT-dependent gene expression (Yuan et al., 2008; Wang et al., 2013). FIT also interacts with subgroup IVa bHLHs, triggering the degradation of FIT *via* the 26S proteasome pathway (Cui et al., 2018). Alongside FIT, POPEYE (PYE)/bHLH47 is another major transcriptional regulator of iron deficiency response in Arabidopsis (Long et al., 2010). *PYE* is expressed under iron deficiency and negatively regulates its target genes, which include those involved in iron translocation, storage, and assimilation. ILR3/bHLH105 plays a dual role in iron homeostasis; depending on the heterodimer it forms, ILR3 activates *PYE* expression (Zhang J. et al., 2015) or represses PYE-target genes (Tissot et al., 2019). UPSTREAM REGULATOR OF IRT1 (URI)/bHLH121 directly or indirectly positively regulates multiple iron homeostasis genes of the bHLH network (Kim et al., 2019; Gao et al., 2020; Lei et al., 2020). URI controls nearly half of iron-regulated genes, including both FIT-dependent and independent genes (Kim et al., 2019; Gao et al., 2020). Although *URI* expression is not iron-regulated, phosphorylation of its protein stabilizes it to form heterodimers with subgroup IVc bHLH transcription factors and activate subgroup Ib bHLH genes under iron deficiency (Kim et al., 2019). Upon iron re-supply, phosphorylated URI is targeted by the E3 ligase BRUTUS (BTS) and subjected to proteasome-mediated degradation. The IRONMAN/FE-UPTAKE-INDUCING PEPTIDE (IMA/FEP) peptides also positively regulate iron deficiency response in Arabidopsis (Grillet et al., 2018; Hirayama et al., 2018) by sequestering BTS to prevent degradation of bHLH105/bHLH115 and activate iron uptake (Li et al., 2021).

Responses to iron deficiency in grasses also utilize several bHLH transcription factors (Gao and Dubos, 2021). OsFIT/OsbHLH156 positively regulates Strategy II-related genes such as those involved in MA biosynthesis and also regulates *OsIRT1*, a Strategy I-related gene (Liang et al., 2020; Wang et al., 2020). OsIRO2 interacts with OsFIT to promote its nuclear localization and positively regulate iron uptake by OsIRT1 (Ogo et al., 2006, 2007; Liang et al., 2020; Wang et al., 2020). OsIRO3/OsbHLH63 represses iron deficiency response possibly *via* antagonizing OsIRO2 to avoid iron overload by limiting iron uptake (Zheng et al., 2010; Gao and Dubos, 2021).

## Iron homeostasis and histone modification

Each nucleosome consists of an octameric complex of histones subjected to a wide range of post-translational modifications. These modifications are reversible but are controlled by many histone modifying enzymes and play key roles in regulating chromatin structure and transcription (Bannister and Kouzarides, 2011; Zhang T. et al., 2015). Multiple iron homeostasis genes in Arabidopsis have been found to be controlled by histone modifications as discussed in this section.

### H3K4me3

H3K4me3, the trimethylation of histone 3 lysine 4, generally leads to gene activation (Liu et al., 2010; Xiao et al., 2016). Using a forward genetics screen in Arabidopsis, Singh et al. (2021) identified a regulator of iron deficiency response, NON-RESPONSE TO Fe-DEFICIENCY2 (NRF2). In Arabidopsis, NRF2 is known as EARLY FLOWERING8 (ELF8), which regulates *FLOWERING LOCUS C (FLC)* expression *via* H3K4me3 (He, 2009). NRF2/ELF8 belongs to the trithorax group (TrxG) methyltransferases that modify histones to activate genes *via* relaxing chromatin structure and serve as antagonistic regulators of polycomb group proteins (Schuettengruber et al., 2011).

Under iron deficiency, AtNRF2/ELF8 is required for *AtGRF11* expression as it modulates H3K4me3 levels at its transcription start site (Singh et al., 2021). While AtGRF11 does not directly interact with *AtFIT*, it acts downstream of NO to induce *AtFIT* expression in iron deficient roots (Singh et al., 2021). In the *nrf2* mutant, AtGRF11-regulated iron uptake was repressed and iron transport and storage genes were downregulated. The mutant normally induced NO under iron deficiency, suggesting that the repression of *AtGRF11* was solely responsible for the regulation of iron uptake genes (Singh et al., 2021).

H3K4me3 also likely regulates the expression of iron storage genes *AtFERRITIN1 (FER1)*, *AtFER3*, and *AtFER4* in iron sufficient seedlings (Tissot et al., 2019). At the promoter regions of these ferritin genes, activation marks such as H3K4me3 and histone 3 lysine 9 acetylation (H3K9ac) were detected in seedlings grown under iron sufficient conditions, whereas H3K27me3 was not present based on analysis of publicly available epigenome profiles (Tissot et al., 2019; Park et al., 2020).

### H3K27me3

The trimethylation of histone 3 lysine 27 (H3K27me3) is typically associated with gene repression; it spreads along the chromatin, resulting in compaction and the silencing of targeted genes (Liu et al., 2010; Xiao et al., 2016). H3K27me3 is catalyzed by Polycomb Repressive Complex 2 (PRC2) (Margueron and Reinberg, 2011). CURLY LEAF (CLF) is a predominant methyltransferase of the core PRC2 complex (Chanvivattana et al., 2004; Schubert et al., 2006; Zhang et al., 2007). In Arabidopsis, H3K27me3 was found to modulate the expression of FIT-dependent genes by directly targeting their loci (Park et al., 2019). Under iron deficiency, the expression of FIT-dependent genes, such as *AtFIT, AtIRT1*, *AtFRO2*, and *AtF6’H1*, was significantly higher in *clf* than in wild type roots, and their transcript levels inversely correlated with H3K27me3 deposition on their loci (Park et al., 2019). However, expression of PYE-dependent genes was not significantly affected (Park et al., 2019). Transcriptomic analysis revealed that transcript levels of FIT-dependent genes were consistently higher in *clf* even under iron-sufficient conditions where FIT-dependent gene expression is extremely low, but the lack of the H3K27me3 mark in iron sufficient *clf* mutants was not sufficient to fully induce FIT-dependent genes when upstream iron-deficiency signals were not present. In iron-deficient conditions, the residual H3K27me3 on FIT-dependent genes may be attenuating the induction of iron acquisition genes to limit their maximum induction to prevent plants from iron-induced cytotoxicity.

H3K27me3 was also implicated to play a role in iron translocation from roots to shoots in Arabidopsis (Park et al., 2020). Iron-deficient *clf* mutants accumulated less iron in the roots than the shoots, but *clf* seedlings still had higher levels of iron compared to wild type. This phenotype and the higher expression of iron acquisition genes in *clf* roots (Park et al., 2019) suggest that *clf* mutants may still be acquiring more iron without retention in the roots due to greater translocation (Park et al., 2020). Indeed, the expression of *AtYSL1*, which encodes an iron-NA transporter involved in supplying iron to sink tissues (Waters et al., 2006), was significantly increased in *clf* compared to wild type and *AtYSL1* was verified to be a direct target of H3K27me3 (Park et al., 2020). *AtIMA1* was also revealed to be a direct target of H3K27me3, but under iron deficiency, H3K27me3 appears to play a limited role in regulating *AtIMA1* expression (Park et al., 2020).

### H4R3sme2

Shk1 binding protein 1 (SKB1) catalyzes the symmetric dimethylation of histone4 arginine3 (H4R3sme2) and regulates diverse biological processes including response to salt stress (Niu et al., 2007; Pei et al., 2007; Wang et al., 2007; Schmitz et al., 2008; Zhang et al., 2011). SKB1-mediated H4R3sme2 also affects iron homeostasis by negatively modulating the expression of Ib subgroup bHLH genes that encode FIT-interacting partners, such as *AtbHLH38/39/100/101*, in response to iron (Fan et al., 2014). While *AtSKB1* expression is not regulated by iron, the level of SKB1 association and H4R3sme2 deposition on the Ib subgroup bHLH loci positively correlated with the iron status of plants. As a result, transcript levels of the Ib subgroup *AtbHLH* genes and its downstream genes including *AtFRO2* and *AtIRT1* that are not direct targets of SKB1 were higher in *skb1* mutants than in wild type roots. Although SKB1 did not affect *AtFIT* expression, transcript levels of *AtFRO2* and *AtIRT1* were not significantly increased in the *skb1 fit1* double mutant, indicating that the negative regulation of iron acquisition genes by SKB1 was dependent on FIT (Fan et al., 2014). The mechanism by which SKB1 perceives iron levels and other environmental signals to determine the degree of H4R3sme2 in specific genes remains to be understood.

### Histone acetylation

Histone acetylation is generally associated with transcriptional activation, in contrast to the more complex effects of histone methylation on gene expression (Berger, 2007). The combined action of histone acetylation and deacetylation is crucial for regulating gene expression (Grunstein, 1997). GENERAL CONTROL NON-REPRESSED PROTEIN5 (GCN5) is responsible for the acetylation of H3K14 and facilitates the acetylation of H3K9 and H3K27, which are required for the expression of a large number of genes (Vlachonasios et al., 2003; Earley et al., 2007; Benhamed et al., 2008).

Xing et al. (2015) reported that AtGCN5 contributes to iron homeostasis by modulating the expression of Arabidopsis *FERRIC REDUCTASE DETECTIVE3 (AtFRD3)*, which encodes a transporter that loads citrate into the xylem to aid translocation of iron-citrate complexes to the shoots (Durrett et al., 2007). AtGCN5 directly binds to the promoters of *AtFRD3* and other iron responsive genes to control H3K9ac and/or H3K14ac levels. In the *gcn5* mutant, iron-related phenotypes similar to those of *frd3* were observed due to significantly decreased H3K9ac and/or H3K14ac deposition at the *AtFRD3* locus and reduced expression of *AtFRD3* (Xing et al., 2015). In the mutants of two histone deacetylases, *hda7* and *hda14*, *AtFRD3* transcript level was increased, providing an example of the coordination between histone acetylation and deacetylation to precisely regulate gene expression (Xing et al., 2015).

## Iron homeostasis and DNA methylation

DNA methylation controls gene expression and contributes to silencing of transposons to maintain genome stability (Law and Jacobsen, 2010; Zhang et al., 2018). In plants, methylation of cytosine occurs in symmetric methylation at CG and CHG, where H represents A, T, or C, and asymmetric methylation at CHH (Matzke and Mosher, 2014; Matzke et al., 2015). While CG and CHG methylations are maintained during DNA replication, CHH methylations are established *de novo* after DNA replication *via* RNA-dependent mechanisms and are frequently found between condensed and relaxed chromatin near highly expressed genes (Gent et al., 2013; Martin et al., 2021).

A recent report suggested that CHH DNA methylation modulates iron deficiency response in rice *via* changing methylation status of genes encoding two major positive regulators of iron deficiency response, *OsIRO2* and *OsbHLH156* (Sun et al., 2021). In this study, widespread hypermethylation, mainly CHH methylation, was detected in rice roots and shoots grown in iron deficient conditions by mapping the DNA methylome at a single-base resolution. Although little correlation was found between CHH hypermethylation and expression of iron deficiency response genes, *OsIRO2* and *OsbHLH156* exhibited CHH hypermethylation and their expression increased under iron deficiency. Furthermore, treatment of 5-aza-2-deoxycytidine (Aza), a DNA methylation inhibitor, and the loss of *OsDRM2*, a key methyltransferase responsible for CHH methylation, resulted in lower expression of *OsIRO2* and *OsbHLH156*, accumulation of less iron, and growth retardment under iron deficiency (Sun et al., 2021). It was speculated that small RNAs might play a critical role as rice acclimates to iron deficiency, as the levels of 24-nt siRNAs increased, whereas transcript levels of canonical RNA-dependent DNA methyltransferases involved in CHH methylation did not change under iron deficient conditions (Sun et al., 2021).

In barley, iron deficiency led to a general reduction of CG methylation, but the overall methylation and demethylation status was not recovered after iron resupply (Bocchini et al., 2015). Further studies are necessary to understand the extent to which DNA methylation or demethylation is maintained upon changes in iron conditions and mechanisms therein.

DNA methylation status was also proposed to be involved in feedback mechanisms between iron status and tolerance to cadmium stress (Fan et al., 2020). Arabidopsis plants exposed to cadmium stress expressed lower levels of the three DNA demethylase genes *AtROS1/DML2/DML3 (RDD)* and exhibited increased global DNA methylation that resembled the methylation profile of *rdd* triple mutants (Fan et al., 2020). The *rdd* mutants were more tolerant against cadmium stress and accumulated more iron in the shoots by expressing higher levels of iron deficiency response genes than wild type. However, inadequate iron supply abolished cadmium tolerance in *rdd* mutants (Fan et al., 2020).

## Conclusion and perspectives

**FIGURE 1:**
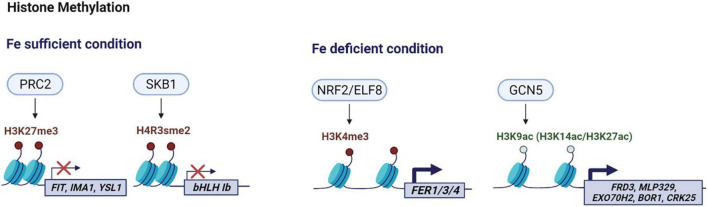
Schematic overview of histone modifications involved in iron homeostasis in Arabidopsis. Under iron sufficiency, PRC2-mediated H3K27me3 induces chromatin condensation in Arabidopsis, resulting in gene silencing of the target genes such as *AtFIT*, *AtIMA1* and *AtYSL1*. AtSKB1-induced H4R3sme2 also suppresses *AtbHLH1b* transcripts when iron is sufficient. Under iron deficiency, AtNRF2/ELF8 catalyzes the trimethylation of H3K4 generating H3K4me3, and AtGCN5 acetylates H3K9 producing H3K9ac and facilitates the generation of H3K14ac and H3K27ac to activate corresponding target genes. The color scheme denotes methylation (red), acetylation (light green), histone (light blue). This figure was created with BioRender.com.

**FIGURE 2:**
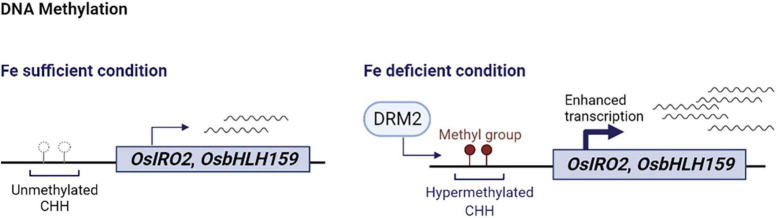
Schematic summary of DNA hypermethylation and iron deficiency response in rice. Under iron sufficient conditions, the CHH sequences of *OsIRO2* and *OsbHLH159* promoters remain unmethylated and basal levels of *OsIRO2* and *OsbHLH159* are expressed. Upon iron deficiency, hypermethylation of CHH nucleotides on the promoters of *OsIRO2* and *OsbHLH159* by DRM2 leads to activation of the expression of the corresponding downstream genes in response to iron deficiency. This figure was created with BioRender.com.

Increasing evidence has shown that iron homeostasis gene expression is affected by histone modification (Figure 1) and DNA methylation (Figure 2). Such chromatin-based regulation is critical during iron deficiency and allows to fine-tune iron homeostasis in plants. Given that chromatin-based regulation is a dynamic process, it will be important to understand the mechanistic details regarding changes in histone modification or DNA methylation in response to changes in iron status. Research to date has mainly focused on iron deficiency and little is known about the effect of iron overload on chromatin remodeling *via* histone modification or DNA methylation. Global changes in H3K9me2 and H3K4me3 levels under high iron stress conditions were detected in the proximal root meristem in rice (Polosoro et al., 2019), but further studies are needed to understand the underlying mechanisms and the biological implications. Furthermore, it will be necessary to integrate large scale datasets of various histone modifications, DNA methylation, and the combinatorial effect of different modifications, as well as comparative analyses of transcriptomics and epigenetics of specific cell-types or at a single cell level. Although chromatin-based regulation is an integral part of epigenetics, some chromatin modifications are not heritable or considered epigenetic (Eichten et al., 2014). Thus, transgenerational studies to determine the heritability of chromatin modifications in response to iron will lead to insightful information. Considering the growing evidence that reveal the significance of dynamic adjustment in chromatin structure and subsequent transcriptional changes in response to nutritional status, a clear understanding of chromatin-based iron homeostasis is necessary for a comprehensive understanding of iron homeostasis. Such efforts will contribute insights toward developing crops with improved nutritional profiles and enhanced tolerance to undesirable conditions in the long run.

## Author contributions

JS primarily wrote the initial draft of the manuscript. ZY and YW contributed to the manuscript writing and generated the figures. JL and JJ conceived the idea and made final edits. All authors contributed to the article and approved the submitted version.
